# The Mechanical and Clinical Properties of Customized Orthodontic Bracket Systems—A Comprehensive Review

**DOI:** 10.3390/jfb15100299

**Published:** 2024-10-07

**Authors:** Issa Elabed, Zhong Zheng, Yu Zhang, Chun-Hsi Chung, Chenshuang Li

**Affiliations:** 1 School of Dental Medicine, University of Pennsylvania, Philadelphia, PA 19104, USA; 2 Department of Periodontics, School of Dental Medicine, University of Pennsylvania, Philadelphia, PA 19104, USA; 3 Department of Preventive and Restorative Sciences, School of Dental Medicine, University of Pennsylvania, Philadelphia, PA 19104, USA; 4 Department of Orthodontics, School of Dental Medicine, University of Pennsylvania, Philadelphia, PA 19104, USA

**Keywords:** 3D printing, CAD/CAM, mechanical properties, clinical performance, metal, zirconia, resin

## Abstract

The rise of computer-aided design and computer-aided manufacturing (CAD/CAM) and 3D printing technologies in orthodontics has revolutionized the development of customized labial and lingual bracket systems with a variety of materials, which offer potential advantages over traditional orthodontic brackets. To highlight the current state of knowledge regarding the mechanical and clinical properties of CAD/CAM and 3D-printed custom bracket systems, we conducted a comprehensive search across the PubMed, Embase, Cochrane Library, Web of Science, and Scopus databases to identify relevant articles published before April 2024. Mechanical (including fracture toughness, hardness, modulus of elasticity, frictional resistance, slot accuracy, torque transmission, and shear bond strength) and clinical (including treatment efficiency and duration, cost, and comfort) properties were compared between traditional and customized orthodontic bracket systems in the current review. Our findings suggest that customized brackets have the potential to increase bracket slot precision, reduce treatment time, and offer cost-efficiency. However, it is worth noting that the advantages and disadvantages of customized bracket systems vary depending on the bracket material and the manufacturing methods, warranting comprehensively controlled investigations in the future.

## 1. Introduction

Orthodontics, a field in constant evolution, has been driven by the fundamental goal of optimizing treatment outcomes through bracket accuracy [1]. This goal stems from the desire to correct malocclusion, one of today’s most prevalent dental irregularities affecting speaking, mastication, and a multitude of other daily functional necessities [2]. This complex issue was firstly tackled using non-programmed brackets made by hand in the early 20th century by pioneers such as Edward Angle, who invented the ribbon arch and edgewise appliance [3]. Although innovative for its time and deeply focused on the principles of force and torque, this method was soon superseded by the straight-wire appliance developed by Larry Andrew [4]. The straight-wire appliance embodied a philosophy known as the “six keys to normal occlusion”, which revolutionized the field by using average anatomical values of the population to program specific first, second, and third order prescriptions into the bracket of each tooth. By targeting predefined treatment outcomes, it aimed for functional accuracy, ensuring that orthodontists had a tool that incorporated information such as tip and torque to correct malocclusion according to six crucial keys that could fit most patients in a one-size fit [4].

It was not long before orthodontists began to question the versatility of Andrew’s approach using bracket prescriptions based on the average anatomical values. Given the variations in human dentition, was the one-size-fits-all approach of brackets the most optimal solution for patients? Notably, previous studies had identified remarkable differences in the angulation of the brackets on the tooth surface due to anatomical variation and human error [5], which significantly tempered the bracket’s accurate positioning—the predominant element in the straight-wire appliance’s success. To reduce human error in bracket positioning, the indirect bonding system was developed [6]. However, previous studies have demonstrated that there is no significant difference in bracket placement errors when using either direct or indirect bonding techniques [7,8]. Furthermore, Mietheke et al. stated that intraindividual variation in tooth morphology is larger than the variation between different types of preadjusted appliances [9]. Thus, it is safe to say that although Andrew’s invention markedly advanced the development of orthodontic treatment, it is far from perfect in creating a customized approach without the digital technology available today. During this period of bracket evolution, there was also a transition in the materials from metal to alternatives such as ceramics and polymers, which was a promising step towards improving the aesthetic appearance of the brackets. However, these materials weren’t without their limitations, as brackets made of ceramics exhibited the disadvantages of higher brittleness and friction compared to metal brackets [10,11,12,13,14,15]. On the other hand, the aesthetically attractive plastic brackets introduced by Newman were characterized by low bond strengths, discoloration issues, wing fractures, and slot distortions due to their low stiffness [16,17].

Achieving true customization with conventional brackets remained a challenge until the late 1990s when the pioneer Dirk Wiechmann developed the Incognito (lingual metal bracket) system using computer-aided design and computer-aided manufacturing (CAD/CAM) technology. Wiechmann’s Incognito system is the first custom-milled bracket, providing a new treatment option in addition to traditional brackets made by injection molding [18,19]. By incorporating CAD/CAM technology, it offered individualized brackets for each lingual surface of a tooth, providing solutions to the challenges posed by conventional systems, such as inaccurate bracket positioning, chairside wire-bending, and torque play [20]. This breakthrough also inspired the invention of labial-based CAD/CAM solutions, such as the Insignia bracket system [21]. The integration of CAD/CAM technologies to create lingual and labial brackets marked a shift in the specialty, focusing on the customization of the bracket to accurately match the bracket base with individual tooth anatomy, thereby optimizing force transmission for desired orthodontic movements [1].

More recently, the recognition of the potential of 3D printing, initially introduced by Hideo Kodama in the 1980s, has also promised a future where orthodontic treatment can focus on individualized care [22]. Emerging from Kodama’s technology is the introduction of lingual and labial 3D-printed metal, ceramic, and plastic brackets [1,23]. These advancements have allowed for the direct fabrication of customized brackets via 3D printing, enabling clinicians to design and create patient-specific brackets to their choice [24]. This may show significant clinical potential for shortening the treatment duration, as well as for reducing white spot lesions if these brackets’ properties exceed those of their traditional counterparts [25]. 3D-printed and customized brackets can either be fabricated in-house, such as the UBracket’s CAD system, or through commercialized systems, such as KLOwen, Braces on Demand, and LightForce [23,24,26].

The advancements in bracket customization represent a significant step forward in orthodontics. However, a deeper understanding of these new technologies is imperative before they become standard practice. Analyzing the clinical efficacy of customized milled or printed brackets necessitates thoroughly examining their properties, impacts on treatment duration, cost effectiveness, and aesthetic outcomes compared to traditionally used injection-molded metal brackets. Therefore, this review aims to compare the properties of newly customized CAD/CAM and 3D-printed bracket systems with traditional brackets based on the currently available literature, provide a clear outline of the existing research on customized brackets, and highlight the unaddressed areas that require future studies.

## 2. Materials and Methods

A comprehensive literature search was conducted in core databases, including PubMed, Embase, Cochrane Library, Web of Science, and Scopus, to include all available original studies on customized orthodontic brackets. The search strategy utilized a combination of keywords such as “orthodontic metal brackets”, “3D printed customized brackets”, “CAD/CAM customized brackets”, and several mechanical and clinical properties (as listed in Table 1, Table 2, Table 3 and Table 4) as related to each bracket type. Studies containing relevant data for the review were selected for full-text screening. Studies published as conference abstracts, editorials, opinions, and literature reviews were excluded from this review. There was no restriction on publication time or languages. The literature search was completed in April 2024.

## 3. CAD/CAM Customized Bracket Properties

### 3.1. CAD/CAM Customized Lingual Brackets

Initially introduced to the orthodontic field by Dr. Wiechmann in the early 21st century, CAD/CAM technologies have been pivotal in creating customized lingual brackets [18,19,20]. Unlike aesthetic labial brackets that can be made from various materials such as plastic and ceramic, CAD/CAM lingual brackets are predominately made from metal, such as Incognito’s gold-alloy brackets and Harmony’s metal alternatives [27]. In addition to offering an option that is discreet in appearance, these CAD/CAM customized lingual systems feature a reduced bracket size and the incorporation of mesh bonding pads that better adapt to the lingual tooth surface through digital technologies. Thus, they not only improve patient comfort compared to traditional metal lingual bracket systems but also arguably offer several mechanical advantages over traditional labial bracket systems [25] (Table 1).

**Table 1:** The reported mechanical and clinical properties of CAD/CAM customized metal brackets compared to traditional metal labial/buccal (TMB) and lingual (TML) brackets.

Bracket System (Brands)		CAD/CAM Customized Lingual (Incognito; Harmony)	CAD/CAM Customized Labial (Insignia)
Mechanical Properties	Fracture Toughness	-	-
	Hardness	-	-
	Modulus of Elasticity	-	-
	Frictional Resistance	Higher (compared to both TMB and TML) [28]	-
	Slot Accuracy	Higher (Incognito compared to TML) [29]	-
	Torque Momentum	Higher (Incognito compared to TMB) [30]	-
	Shear Bond Strength	Lower (Incognito and Harmony compared to TMB and Insignia) [31]	Higher (compared to TMB, Incognito, and Harmony) More brackets debond (compared to TMB) [31,32]
Clinical Properties	Treatment Outcome	Less effective in vertical and anteroposterior corrections (Incognito compared to TMB) [33]	Inconsistent conclusions from different studies (compared to TMB)
	Chair Time	Shorter (Harmony compared to TMB) [34]	-
	Treatment Duration	-	Inconsistent conclusions from different studies (compared to TMB)
	Color Stability	-	-
	Discomfort	Less (compared to TML) [35,36]	Inconsistent conclusions from different studies (compared to TMB)
	Cost	Higher (compared to TMB) [37]	Higher (compared to TMB) [38]
	White Spot Lesions	Fewer (compared to TMB placed buccally) [39]	-

#### 3.1.1. Mechanical Properties

Overall, compared to labial systems, lingual systems have shorter inter-bracket distance, which results in increased archwire stiffness and frictional forces [40]. When comparing lingual customized brackets (such as Incognito and Harmony) with lingual traditional milled brackets systems (STb), the customized systems have higher frictional values, with the Harmony system topping the list (10% higher than STb) [28].

Regarding slot precision, a study by Demling et al. showed that customized Incognito brackets had higher slot precision than lingual traditional milled seventh-generation and STb systems [29]. For 0.018 brackets systems, Incognito’s slot width measured average was 0.0181 inches, while seventh-generation and STb brackets had an average of 0.0184 inches [29]. These slot differences should be considered in orthodontic treatment, as they can result in improved clinical torque. For example, Sifakakis et al. reported that customized Incognito brackets caused 23% higher torque momentum compared to traditional labial metal Gemini (3M Unitek, Monrovia, CA, USA) brackets [30].

The bonding properties of lingual customized bracket systems have also been evaluated and compared to labial traditional metal brackets. Although lingual customized brackets have lower shear bond strength than labial traditional metal brackets (5.09 MPa vs. 6.73 MPa), the bonding area of lingual customized brackets is much larger than that of traditional labial brackets, which may still provide clinically reliable bonding strength when using lingual customized brackets. In fact, in an in vitro test with brackets placed by indirect bonding, lingual customized brackets had much higher debonding force (169 N) compared to labial traditional milled brackets (62 N) and labial customized brackets (Insignia, 118 N) [31]. In addition, when evaluating based on the failure mode [41], Incognito’s alloy brackets showed the highest proportion of resin remaining on the bracket after debonding, indicating a strong bond at the bracket-adhesive interface, followed by Harmony/self-ligating custom lingual brackets [31]. However, it is essential to note that in this study [31], Incognito brackets were treated with sandblasting and silane coating, which arguably enhances bonding ability [42,43]. Thus, the findings may stem from the bonding protocol but not the bracket itself.

#### 3.1.2. Clinical Properties

Placing a bracket lingually and near the centers of resistance of the teeth alters the force vectors applied, resulting in different clinical effects compared to traditional labial systems [44]. Previous studies have demonstrated that traditional labial metal, traditional lingual, and customized lingual brackets provided different labiolingual forces on the sagittal plane, while custom and traditional lingual brackets shared some similarities [45]. Such force differences introduced different efficiency in correcting vertical and anteroposterior displacement during initial alignment, with Incognito alloy brackets less effective than traditional metal labial brackets [33]. However, through careful planning and custom bracket design, effective forces can be applied in the anterior region with Incognito brackets, and final alignment results that are comparable to labial traditional systems can be achieved [37].

Customized lingual brackets have also been associated with a reduced chair time of around 8 min, especially when using self-ligating techniques like the customized Harmony bracket systems [34]. In addition, patients with CAD/CAM lingual brackets reported fewer restrictions and disturbances in speech, chewing, and biting compared to the traditional lingual Ormco prefabricated bracket group [35,36]. Thus, customized fit significantly improved the comfort level of patients, which might be due to the thinness of the customized brackets. Furthermore, customized lingual brackets have been linked to a lower incidence of white spot lesions [39,46]. Studies reported that white spot lesions developed 4.8 times more often on buccal surfaces when brackets were placed buccally than on lingual surfaces when bonded with customized lingual brackets [39,46]. This is possibly due to bracket positioning and material properties that discourage enamel demineralization compared to labial brackets.

#### 3.1.3. Summary

Overall, CAD/CAM customized lingual brackets, as a relatively broadly used customized system, offer the aesthetic advantages of traditional lingual brackets while enhancing patient comfort by adapting to individual tooth anatomy. On the other hand, their application requires a thorough understanding of biomechanical principles and lingual forces before clinical use, mainly due to the high friction and poor control over vertical and anteroposterior corrections.

### 3.2. CAD/CAM Customized Labial Brackets

CAD/CAM customized labial brackets are a significant advancement in orthodontics, moving away from traditional approaches towards an individualized approach by building on the foundation of Weichmann’s CAD/CAM lingual bracket system (Table 1). In terms of labial systems, Insignia is a popular system for customized milled labial brackets [1,47]. The system begins by converting detailed impressions into a digital model of the patient’s malocclusion. Orthodontists can then use computer-aided design (CAD) software to customize every aspect of the treatment, from designing each customized bracket to specific torque, tipping, and intrusion and extrusion movements, as well as to customize wire bending, ensuring efficient movement [48]. Cases are then sent out of the office to technicians who engineer each bracket precisely through milling and welding to create each self-ligating metal bracket. Once the process is complete, the bracket set is then sent over to the clinician for indirect bonding placement [49].

#### 3.2.1. Mechanical Properties

Labial customized Insignia brackets have nearly double the stem lengths, i.e., the distance between the bracket wings and bracket base, than conventional labial metal brackets and lingual Incognito brackets, which could influence their shear bond strength [31,50]. Regarding mechanical properties, previous studies suggested that Insignia brackets have higher shear bonding strength (SBS) (9.99 ± 3.36 MPa) than traditional labial metal brackets (6.73 ± 1.36 MPa) [31,32]. However, contradicting the findings in SBS, the failure mode showed that Insignia brackets had 17% more brackets with adhesive left on the tooth after debonding than that observed on Incognito and are comparable to traditional labial metal brackets [32]. In addition, more patient complaints and more bracket loosening in the customized group were reported in three different studies [49,51,52], despite the high SBS reported in the in vitro test.

#### 3.2.2. Clinical Properties

Treatment duration and outcomes are prominently debated topics when comparing CAD/CAM customized orthodontic brackets to traditional ones. Since CAD/CAM systems demonstrate a technological advantage in terms of reducing the need of bracket repositioning, the Insignia system advertised a 37% reduction in treatment time and 15% reduction in patient visits [38], which was proven by some research groups [1,53,54]. For example, a case report by Choi et al. demonstrated that utilizing the CAD/CAM customized labial bracket system in orthognathic–orthodontic cases could shorten the total treatment time to 16 months [55]. Noticeably, this claim is not necessarily supported by other studies: some studies showed that CAD/CAM customized labial systems could reduce the total treatment duration by 3 months compared to indirect bonded prefabricated self-ligating systems, but not necessarily reduce appointment numbers [2,5,56], while other studies suggested that CAD/CAM customized labial systems could not reduce the total treatment duration at all [49,51,52]. Regarding treatment outcomes, Khan et al. reported that the Insignia system has a clinical efficiency similar to that of non-customized systems, with no difference in lowering the peer assessment rating (PAR) score [2]. Similarly, Jackers et al. found that the CAD/CAM customized bracket system and the indirect bonding self-ligating bracket system resulted in the same quality of treatment [56]. On the contrary, Liu et al. found that the personalized brackets led to increased dental alignment difficulties, such as midline discrepancies and molar buccal occlusion [49].

Besides the two hot topics discussed above, inconsistencies were observed in studies evaluating patients’ comfort levels. Khan et al. reported that the CAD/CAM customized labial system is associated with less pain and greater patient comfort; meanwhile, Hurst and Penning found an increase in patient complaints compared to non-customized systems [2]. Therefore, broader studies with suitable control in the future are warranted to accurately assess the clinical properties of CAD/CAM custom labial brackets.

#### 3.2.3. Summary

Although CAD/CAM customization is a step forward in customized orthodontics, evaluating the mechanical and clinical properties of CAD/CAM customized labial brackets is in its infancy. Contradictions observed in the currently available research necessitate more benchtop and chairside assessments of the CAD/CAM customized labial bracket system.

## 4. 3D-Printed Customized Bracket Properties

### 4.1. 3D-Printed Customized Brackets: Metal

Traditional metal brackets have been the foundation of many orthodontic treatments for over a century. Historically, these metal orthodontic brackets were manufactured using standard methods such as investment casting and injection molding. However, the advent of 3D printing has revolutionized the industry by enabling the production of custom-made metal brackets for orthodontic treatments.

Compared to traditional methods, 3D-printed customized metal bracket printing employs an additive manufacturing process [57] in which layers of material are carefully added by a printer to create a final customized bracket [57]. This process is distinct from printing custom ceramic and resin brackets, as it utilizes direct metal laser sintering with stainless steel and titanium alloys, which were first introduced in the 1990s [58,59]. Consequently, 3D-printed metal customized brackets exhibit different mechanical and clinical properties from traditional bracket systems (Table 2).

**Table 2:** The reported mechanical and clinical properties of 3D-printed customized metal brackets compared to traditional metal buccal (TMB) brackets.

Bracket System		3D-Printed Metal
Mechanical Properties	Fracture Toughness	-
	Hardness	-
	Modulus of Elasticity	-
	Frictional Resistance	-
	Slot Accuracy	Higher (compared to TMB); minor bumps found on slot surface [60]
	Torque Momentum	-
	Shear Bond Strength	Lower (compared to TMB) [60]
Clinical Properties	Treatment Outcome	-
	Chair Time	-
	Treatment Duration	-
	Color Stability	-
	Discomfort	-
	Cost	$7 per bracket and 90 min to print both arches [60]
	White Spot Lesions	-

#### 4.1.1. Mechanical Properties

One of the most advantageous aspects of 3D metal printing is the ability to design undercuts directly into the bracket’s base during the CAD process. By replacing the standard mesh with purposely designed undercuts, 3D metal printing improves the bracket’s retention, allowing for a one-piece bracket design and thus streamlining the production process [60].

Slot sizes can vary significantly between brands and batches among traditional metal brackets, posting a major manufacturing obstacle. Independent research by Cash and Kusy revealed that conventional metal bracket slots were either 16% oversized or 15% undersized compared to their indication [61,62]. On the contrary, a study showed that 3D printing can achieve a higher accuracy in the slot dimensions of the customized metal bracket. For example, for metal brackets indicated as having a 0.022-inch slot size, the slot size of 3D-printed brackets was 0.0221 inches, while the slot size of the conventional brackets was 0.0246 inches [60]. The increased slot precision might lead to better tooth positioning, improved torque control, increased treatment efficiency, reduced “third order play”, and potentially shortened treatment durations, which should be explored in future studies [63]. Nevertheless, it is important to note that flaws such as minor bumps on the slot surface were observed in 3D-printed brackets, which cannot be addressed by the polishing process [60]. Therefore, additional investigation is needed to find better solutions.

A study on the SBS of customized metal brackets showed that the bond strength of 3D-printed customized metal brackets was measured at 17.96 ± 2.01 MPa, while the conventional metal Damon and Ti-Orthos brackets were measured at 23.19 ± 7.61 MPa and 19.22 ± 5.11 MPa, respectively [60]. This indicates that, although slightly lower than traditional metal brackets, 3D-printed customized metal brackets can still provide a strong bond to the tooth.

#### 4.1.2. Clinical Properties

For customized metal braces, orthodontists can use specialized software, such as that provided by KLOwen, which allows for customized prescription of each bracket. For orthodontists who own a 3D printer, such as the Mlab cusing metal printer, the in-house printing process for a set of 3D-printed customized metal brackets will take about 90 min and cost an average of $7 per bracket [60]. For orthodontists who do not have an in-house printer, companies like KLOwen offer full lab service with a two-week turnaround [64].

#### 4.1.3. Summary

The limited data available suggest that 3D-printed customized metal brackets have potential advantages over traditional ones. Since there is only one published master’s thesis focused on this topic, extensive investigations are required to assess this new technology comprehensively.

### 4.2. 3D-Printed Customized Brackets: Ceramic

Thanks to technological developments, ceramic brackets can now be 3D-printed, potentially combining the benefits of customization and aesthetics. With several ceramic materials that are feasible for 3D printing, it is critical to compare their advantages and drawbacks to determine their suitability for bracket manufacturing (Table 3).

**Table 3:** The reported mechanical and clinical properties of 3D-printed customized ceramic brackets compared to traditional metal buccal (TMB) brackets, traditional polycrystalline alumina ceramic buccal (TPCB) brackets, and traditional monocrystalline alumina ceramic buccal (TMCB) brackets.

Bracket System		3D-Printed Ceramic		
		Polycrystalline Alumina	Lithium Disilicate	Zirconia
Mechanical Properties	Fracture Toughness	Lower (compared to TPCB) [65]	-	Higher (compared to TPCB and 3D-printed alumina) [65]
	Hardness	Lower (compared to TPCB) Higher (compared to 3D-printed zirconia) [65,66]	-	Lower (compared to TPCB and 3D-printed alumina) [65,67]
	Modulus of Elasticity	-	-	-
	Frictional Resistance	-	Lower (compared to TPCB) Higher (compared to TMCB) [68]	-
	Slot Accuracy	-	No obvious defects (compared to TPCB) [68]	-
	Torque Momentum	-	-	-
	Shear Bond Strength	-	Similar (compared to TMB) Lower (compared to TPCB) [68]	-
Clinical Properties	Treatment Outcome	Fewer loose brackets and superior final tooth alignment (compared to TMB) [69]	-	-
	Chair Time	-	-	-
	Treatment Duration	Shorter (compared to TMB) [69]	-	-
	Color Stability	-	Less (compared to TPCB) [68]	-
	Discomfort	-	-	-
	Cost	Higher (compared to TMB) [69]	-	$1000 per 1 L slurry (1.5 mL required per 24 brackets) [21,26]
	White Spot Lesions	-	-	-

#### 4.2.1. 3D-Printed Customized Ceramic Brackets: Polycrystalline Alumina

One of the commercialized 3D-printed polycrystalline alumina brackets is the LightForce system. According to the patent file, LightForce brackets are manufactured by digital light processing (DLP) of ceramics additive manufacturing (AM) technology, followed by a thermal debinding and sintering step, which may provide better surface quality, object resolution, and mechanical properties over brackets fabricated by selective laser sintering/melting (SLM) [70].

##### Mechanical Properties

While the aesthetics of ceramic brackets are superior to those of metal brackets, they have long been criticized for their brittleness. Orthodontists often face challenges with bracket wing fractures, which complicate the removal process and increase overall treatment costs. Regrettably, Polychronis et al. reported that 3D-printed customized polycrystalline alumina brackets exhibited lower fracture toughness than conventional polycrystalline alumina ceramic brackets (4.44 ± 0.30 MPa M^½^ of 3D-printed vs. 5.30 ± 0.48 MPa M^1/2^ of Clarity) [65], which would be clinically unfavorable due to a higher risk of bracket fracture.

On another hand, polycrystalline alumina brackets in the lower arch could cause enamel abrasion in those with deep bites or bruxism due to enamel hardness being 8–10 times lower than alumina brackets, which should be noted during the treatment of certain patient populations [71,72]. LightForce’s 3D-printed customized polycrystalline alumina brackets has lower Vickers hardness value than Clarity’s traditional polycrystalline alumina brackets (1840 ± 38 HV of LightForce vs. 2000 ± 49 HV of Clarity) [65,66]. However, it is still significantly higher than that of the enamel.

##### Clinical Properties

Waldman et al. found that, compared to conventional metal brackets, Lightforce brackets reduced emergency visits caused by loose brackets by 41% and decreased the total treatment time by 45% [69]. Regarding the treatment outcome, the PAR index showed nearly equal clinical results for final tooth alignment and optimal occlusion in the LightForce (PAR score: 5, nearly optimal) and traditional metal bracket groups (PAR score: 7, clinically acceptable) [69].

##### Summary

Due to their relative recentness, limited information is available regarding the mechanical and clinical properties of 3D-printed customized polycrystalline alumina brackets, necessitating extended research. Nonetheless, customized improvements in alumina brackets offer a potentially future for orthodontic therapy.

#### 4.2.2. D-Printed Customized Ceramic Brackets: Lithium Disilicate

Lithium disilicate, used for e.max crown manufacturing, provides another option for 3D-printed customized brackets via DLP, heat pressing, and sintering, and ruby oilstone slices with 0.022 in thickness were used to polish the slot of bracket until a smooth-looking slot was achieved [68]. As lithium disilicate has respectable flexural strength and moderate fracture toughness values [68,73,74], 3D-printed customized brackets that contain it may offer an advantage in terms of avoiding bracket wing fracture. The relatively low hardness of lithium disilicate brackets may show advantage in avoiding enamel abrasion compared to their polycrystalline alumina counterparts [75].

##### Mechanical Properties

The frictional properties of customized brackets play a critical role in orthodontics and sliding mechanics [76]. The frictional resistance of 3D-printed customized 0.022 in slot IPS lithium disilicate brackets on 0.018 × 0.025 in stainless steel archwires was found to be 14% lower than that of 0.022 in slot Clarity polycrystalline alumina ceramic brackets but 57% higher than that of 0.022 in slot Inspire monocrystalline alumina ceramic brackets when using elastic ligation, and it was found to be similar to that of Clarity polycrystalline alumina ceramic brackets and 48% higher than that of Inspire monocrystalline alumina ceramic brackets when using stainless steel ligation [68].

Regarding surface and slot morphology, Yang et al. demonstrated through scanning electron microscopy (SEM) examination that there were no apparent differences between the slots of 3D-printed customized IPS lithium disilicate brackets and conventional ceramic brackets [68].

Orthodontics relies on bonding, which traditional ceramic brackets obtain via mechanical retention or silane coupling [77,78]. Yang et al. found that hydrofluoric acid etching on the base surface of customized lithium disilicate brackets may improve clinical bonding [68]. Particularly, the SBS of 3D-printed customized IPS lithium disilicate brackets was 10.21 ± 2.30 MPa with hydrofluoric acid etching protocol, which was similar to that of conventional metal brackets (10.09 ± 1.07 MPa) but lower than that of conventional ceramic brackets such as Clarity (14.02 ± 2.00 MPa) before silane treatment [68]. Moreover, silane treatment increased the SBS of 3D-printed customized IPS lithium disilicate brackets to 15.16 ± 2.67 MPa [68]. However, the bonding failure mode evaluations showed that 3D-printed customized lithium disilicate brackets had higher adhesive remnant index (ARI: 3) than Inspire (ARI: 2.4), Clarity (ARI: 2.9), and Damon/conventional metal brackets (ARI: 1.8) [68]. The high ARI score indicated that there was more adhesive left on the tooth surface instead of on the bracket base of 3D-printed customized IPS lithium disilicate brackets compared to that of other brackets, which is an unfavorable debonding pattern [79]. Silane treatment decreased the average ARI score of 3D-printed customized lithium disilicate brackets to 2.3.

##### Clinical Properties

Although multicolored and clear materials can be used to make 3D-printed customized IPS lithium disilicate brackets to appeal to the patient [68], long-term color stability remains an issue. Yang et al. found that 3D-printed customized lithium disilicate brackets could be darkened to a yellow color by natural light and tended to be more opaque than commercial brackets [68]. If future development addresses this color stability issue, this type of bracket could not only function optimally as a customized tool but also meet patient’s aesthetic needs by being available in a variety of shades.

##### Summary

Currently available studies suggest that lithium disilicate holds some favorable mechanical properties compared to polycrystalline alumina disilicate [75]. Nonetheless, only a few mechanical properties have been tested on 3D-printed customized lithium disilicate brackets. Additionally, there is a lack of information concerning 3D-printed customized lithium disilicate brackets on orthodontic treatment outcome and intraoral color stability, warranting further investigation.

#### 4.2.3. 3D-Printed Customized Ceramic Brackets: Zirconia

Orthodontists can also create 3D-printed customized zirconia bracket prescriptions via computer-aided design (CAD) software such as Ubrackets [26,80] and DLP technology, followed by debinding and sintering in a Shenpaz SintraPRO sintering unit [65]. By adapting a more precise fit to the tooth anatomy and maximizing force application, 3D-printed customized zirconia brackets may expedite orthodontic procedure times.

##### Mechanical Properties

A study by Polychronis et al. showed that 3D-printed customized zirconia INNI-CERA A2 brackets were 20–30% more fracture-resistant (tougher) than traditional polycrystalline alumina ceramic brackets (Clarity) and 3D-printed customized polycrystalline alumina LightForce brackets [65]. The enhanced toughness of 3D-printed customized zirconia brackets (6.62  ±  0.61 MPa m^1/2^) could mitigate the occurrence of wing fractures, a common issue associated with the brittle nature of other ceramic materials [10,81].

It is important to note that 3D-printed customized zirconia brackets exhibit superior toughness to other ceramics, such as alumina, but possess relatively lower hardness. For example, Polychronis et al. reported that the Vickers hardness of traditional polycrystalline alumina ceramic brackets and LightForce’s 3D-printed customized polycrystalline alumina brackets were 37% and 22% higher, respectively, than 3D-printed customized zirconia brackets (1261  ±  39 HV) [67]. The lower hardness of 3D-printed customized zirconia brackets may be advantageous in preventing enamel abrasion. However, it could also lead to bracket wear from arch wires, reducing slot accuracy and necessitating replacement during treatment [82,83].

##### Summary

It is crucial to thoroughly assess the mechanical and clinical properties of 3D-printed customized zirconia brackets. From a technical standpoint, it is also essential to ensure a homogenous zirconia slurry is used for bracket printing, considering that a heterogenous zirconia slurry could cause bracket instability and shrinkage during sintering [80]. Future studies should also explore whether using different types of software or 3D printers may manufacture brackets with varying properties and accuracy.

### 4.3. 3D-Printed Customized Brackets: Plastic/Resin

Plastic/resin is a major type of material used in 3D printing in a wide range of areas. With the adoption of 3D printing technology in orthodontics, 3D-printed plastic/resin customized brackets have been broadly investigated as well (Table 4). It is necessary to declare the distinctions and similarities among these customized plastic brackets and compare them with traditional metal, ceramic, and plastic brackets.

#### 4.3.1. Mechanical Properties

Concerns have been raised regarding the stability of the wings of 3D-printed customized resin brackets. Among these seven types of resins, permanent crown resin and Sheraprint Ortho Plus are the only two reported in the literature with fracture toughness testing of their 3D-printed brackets. A study by Bauer et al. found that 3D-printed permanent crown resin brackets exhibited adequate fracture toughness [84], with only one out of thirty (1/30) 3D-printed permanent crown resin brackets breaking, compared to nine out of thirty (9/30) traditional polycrystalline alumina ceramic brackets. Conversely, 3D-printed customized resin brackets made of Sheraprint Ortho Plus material demonstrated low fracture toughness with development of cracks in the wings that caused brackets to be replaced during the treatment [1].

**Table 4:** The reported mechanical and clinical properties of 3D-printed customized plastic/resin brackets compared to traditional metal buccal (TMB), traditional polycrystalline alumina ceramic buccal (TPCB), traditional monocrystalline alumina ceramic buccal (TMCB), and traditional plastic buccal (TPB) brackets.

Bracket System		3D-Printed Plastic/Resin						
		Permanent Crown Resin	Temporary Crown Resin	SG Resin	LT Resin	Shark SL	Sheraprint Ortho PLus	GR-10/GR-17.1 Guide Resin
Mechanical Properties	Fracture Toughness	Higher (compared to TPCB) [84]	-	-	-	-	Lower (compared to TMB) [1]	-
	Hardness	Lower (compared to TMB and TPCB) Higher (compared to TPB) [65,85]	Lower (compared to TMB and TPCB) Higher (compared to TPB) [65,85]	-	-	-	-	-
	Modulus of Elasticity	Lower (compared to TMB and TPCB) [85]	Lower (compared to TMB and TPCB) [85]	-	-	-	-	-
	Frictional Resistance	-	-	-	-	Lower (compared to TMB and TPCB) [86,87]	-	-
	Slot Accuracy	Higher (compared to TPB and TMB) [88]	-	-	-	-	-	-
	Torque Momentum	Clinically sufficient (compared to TMB and TPCB) [84]	-	-	-	-	-	-
	Shear Bond Strength	-	-	Clinically sufficient (compared to TMB and TPB) [89]	Clinically sufficient (compared to TMB and TPB) [89]	-	-	-
Clinical Properties	Treatment Outcome	-	-	-	-	-	-	-
	Chair Time	-	-	-	-	-	-	-
	Treatment Duration	-	-	-	-	-	-	-
	Color Stability	-	-	Less (compared to LT Resin) [90]	Higher (compared to SG Resin) [90]	-	-	Unstable [91]
	Discomfort	-	-	-	-	-	-	-
	Cost	$790 per 0.7 L [92]	$499 per 0.7 L [93]	$249 per 1 L [94]	$349 per 1 L [95]	-	-	-
	White Spot Lesions	-	-	-	-	-	-	-

When tested for hardness, Papageorgiou et al. reported that both 3D-printed permanent and temporary crown resin brackets exhibited superior Vickers hardness compared to traditional resin brackets (permanent crown resin brackets: 35 HV, temporary crown resin brackets: 35 HV, and traditional resin brackets: 16.9–19.6 HV) [85]. Since they are harder, 3D-printed customized permanent and temporary crown resin brackets demonstrate greater resistance than conventional plastic brackets, particularly when they come into contact with materials like steel ligations [23]. However, the hardness of 3D-printed customized resin brackets remains significantly lower than that of traditional brackets made from stainless steel or ceramic [85], limiting the clinical applications and effectiveness of 3D-printed customized resin brackets.

The modulus of elasticity, which is correlated to the stiffness of a bracket, is important for effectively transferring loads from the activated arch wire to the tooth. Papageorgiou et al. found that traditional polycrystalline ceramic and metal brackets had a greater modulus of elasticity (62–83 GPa) than 3D-printed customized permanent and temporary crown resin brackets (5.5–5.6 GPa) [85]. Due to their low modulus of elasticity, 3D-printed customized resin brackets may permanently deform even under low forces [96], reducing their clinical treatment efficiency and compromising control over orthodontic tooth movement. Therefore, future research should focus on advancing the heavy filling techniques of 3D-printed resin to enhance their stiffness.

Regarding frictional resistance, Shark SL resin is the only tested and reported 3D-printed plastic/resin material for bracket manufacture in the currently available literature. Hodecker et al. showed that 3D-printed customized Shark SL resin brackets combined with steel arch wires posed the advantage of lower frictional resistance (26% force loss) compared to traditional metal (43% force loss) and traditional ceramic brackets (47% force loss) [86,87].

Meanwhile, only one study was found to assess the slot accuracy of 3D-printed customized permanent crown resin brackets, in which the slot heights of in-house 3D-printed customized permanent crown resin brackets were reported to be 4.30% more accurate than those of commercially available plastic and metal brackets that exhibit excessively higher slot heights than the manufacturers’ indication [88]. It is worth noting that even a tiny difference in slot height can result in increased play between the bracket slot and the arch wire, potentially compromising the torque applied to the teeth [71]. The precise slot size provided by 3D-printed customized permanent crown resin brackets ensures the maintenance of torque momentum [97]. Notably, 3D-printed customized permanent crown resin brackets are able to withstand a crown torque of up to 60 Nmm, which satisfyingly exceeds the accepted threshold and is slightly higher than that of conventional metal and ceramic brackets [84].

The mesh integration present in metal brackets is absent in both 3D-printed and traditional plastic brackets [98]. To enhance retention, manufacturers must incorporate additional structures such as protrusions into the bracket design through CAD. However, due to the low internal strength of 3D-printed customized resin brackets determined based on fragments of the bracket remaining on the enamel surface, intra-bracket failure may occur, rather than the typical debonding at the interface of enamel, adhesive, and bracket base [89]. Among the seven types of resin, only SG resin and LT resin had been tested for bonding strength, and both SG resin (12.09 ±  1.23 MPa with air abrasion, 8.87 ±  0.64 MPa without air abrasion) and LT resin brackets (around 9.90 MPa with air abrasion, around 10.2 MPa without air abrasion) could provide clinically sufficient shear bond strength with or without air abrasion [89]. 

#### 4.3.2. Clinical Properties

The most significant advantage of plastic brackets is their aesthetics. Since the brackets remain in patients’ mouths for a relatively long time, it is crucial to evaluate the stability of their color and transparency. Disappointedly, the current outcome is dissatisfaction. For instance, Haynie et al. evaluated 3D-printed SG resin brackets and LT resin brackets, finding that LT resin was the only material capable of maintaining its color when immersed in wine and exposed to accelerated aging [90]. In addition, Wallach et al. reported significant color and translucency changes in 3D-printed GR-10 and GR-17.1 Guide resin brackets with aging and exposure to both endogenous and exogenous staining sources, undermining their clinical utility [91]. Since both studies were conducted in vitro, further research is needed to determine the applicability of these custom resins in in vivo clinical settings.

#### 4.3.3. Summary

3D-printed customized resin brackets offer certain advantages in mechanical properties and customization options, accompanied by several issues to be improved, such as color instability and plastic deformation. Additionally, given the current lack of studies on the clinical effectiveness of 3D-printed customized resin brackets, researchers need to investigate their impact on orthodontic clinical management, thereby enriching our understanding of their clinical application.

## 5. Conclusions and Future Directions

**Figure 1:**
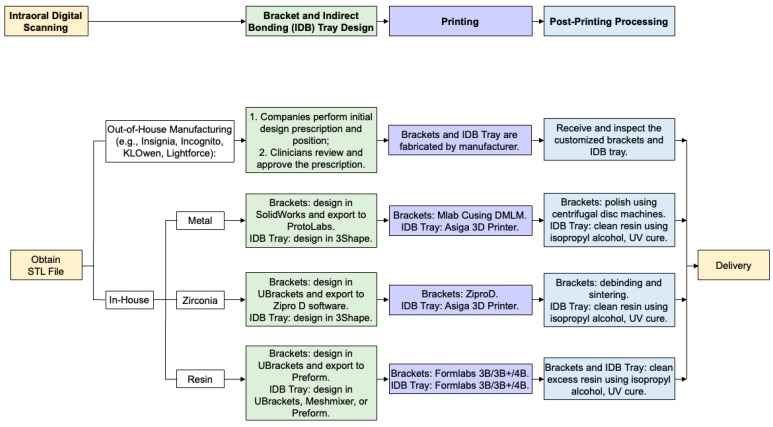
Digital orthodontic workflow of 3D-printed and customized bracket systems for in-house and out-of-house manufacturing.

Two types of customized brackets are currently available: CAD/CAM customized buccal/lingual bracket systems and 3D-printed customized bracket systems. Both systems could be designed with the current digital orthodontic workflow (Figure 1). The key reported features of each type of customized brackets are summarized as below:

(1) CAD/CAM customized lingual brackets: CAD/CAM customized lingual brackets stand out in modern orthodontics because they are discreet and fit each patient’s anatomy precisely. They offer significant improvements in comfort and treatment effectiveness, but it is important to fully understand their biomechanical impacts before use.
(2) CAD/CAM customized labial brackets: Evaluating the mechanical and clinical properties of CAD/CAM customized labial brackets is still in its infancy. Contradictory results about treatment duration and its effectiveness indicate that more research is needed.
(3) 3D-printed metal brackets: 3D-printed metal brackets could possibly blend customization with the efficient properties of the traditional metal bracket. Increased slot accuracy is a promising property, but the evidence is limited.
(4) 3D-Printed Polycrystalline alumina ceramic brackets: The concept behind 3D-printed alumina ceramic brackets is compelling, but they are currently characterized by brittleness and fracture susceptibility. Current research suggests that they speed up orthodontic treatment compared to traditional systems.
(5) 3D-printed lithium disilicate brackets: 3D-printed lithium disilicate brackets may be superior to traditional ceramic brackets given that they have lower frictional resistance. However, the lower color stability compromises the aesthetics—the key feature of ceramic brackets.
(6) 3D-Printed Zirconia brackets: 3D-printed zirconia brackets require extensive research to fully grasp their clinical implications. Initial research points to promising fracture resistance, which minimizes wing fractures, and low hardness, which prevents enamel abrasion compared to conventional ceramics.
(7) 3D-Printed Resin/plastic brackets: Multiple types of resin have been used for 3D-printed resin brackets. But the evaluation of the properties of each type of 3D-printed resin brackets is minimum. Currently, brackets printed with permanent crown resin may hold good clinical potential due to their high fracture toughness, low hardness, high slot accuracy, and clinically sufficient torque momentum.

In summary, while CAD/CAM-customized bracket systems have been studied more extensively, contradictory findings regarding treatment duration and effectiveness necessitate further investigations. On the other hand, the 3D-printed system offers the potential for in-house bracket manufacturing and improved bracket slot accuracy. However, evaluations of various mechanical and clinical properties of 3D-printed brackets made from different materials (metal, ceramic, and resin) are largely lacking. In addition, while representing a significant advancement, the complexity of the design and printing process of 3D-printed brackets is worth noting. Errors may occur throughout the software design process, affecting the final customized bracket. Accurate bracket design and a high-resolution STL file are essential since they determine the precision of bracket positioning and slot accuracy [23]. Furthermore, whether the 3D printing methods and settings (such as speed and orientation) could affect the properties of the brackets also needs to be evaluated. Nevertheless, with the majority of the current available studies being conducted in vitro, challenges related to material durability and long-term wear must be evaluated clinically. More efforts should be given to addressing the obstacles of each printing material, evaluating whether combining different materials could enhance the properties of 3D-printed brackets, and exploring whether the integration of new materials into currently available systems could provide optimized properties of customized brackets.

## Data Availability

The data presented in this study are contained within this article.
